# Supplementary material for: A benchmark for computational analysis of animal behavior, using animal-borne tags
Source: Mov Ecol. 2024 Dec 18;12:78. doi: 10.1186/s40462-024-00511-8 (PMC11654173; doi:10.1186/s40462-024-00511-8)
Supplement: Supplementary file 1 [file 40462_2024_511_MOESM1_ESM.pdf]

Supplemental Information

Time Scales

Animal behavior can be described hierarchically, in which actions are nested into multiple time scales [1, 2]: for example, the human behavior *Walking* may be hierarchically composed of two repeating, shorter time-scale behaviors, the left and right forward steps. For simplicity, in this study we focus on a single non-hierarchical set of annotations per dataset. However, there are multiple time scales represented across the nine ethograms in BEBE. For example, some behavior classes reflect brief, low-level activities (e.g. shaking), whereas some reflect longer, higher-level activities (e.g. foraging, exploration). In order to give a rough quantification of the time scales present in these ethograms, for each dataset we computed the average amount of time an individual spends in a known behavioral state, before it switches to a different known behavioral state or an *Unknown* state. This quantity is reported in Table 2 as the mean annotation duration. The mean annotation duration should only be taken as a rough estimate of the typical duration of a behavioral state, because the annotations in the original studies were not necessarily produced with the intention of measuring onsets and offsets of behavioral states.

For the Polar Bear dataset, to compute mean annotation duration, we had to account for the fact that the video footage used to make annotations was duty cycled. Because of this duty cycling, there are periodic intervals of up to 90 seconds in which annotations are *Unknown*. To account for these *Unknown* intervals, we assumed that if the bear is in the same behavioral state before and after an *Unknown* interval of less than 91 seconds, then the bear was in that behavioral state during the *Unknown* interval. This procedure was only used to compute mean annotation duration, and not to add additional annotations for model training or evaluation.

Color Mapping

For Figure 4, as well as Supplemental Figures S21 and S22, we use the perceptually uniform `inferno` color mapping provided by the MatPlotLib [3] Python package. Before applying the color mapping, we rescale the values in each column (i.e., average scores for a set of models evaluated on the same data) in order to emphasize the relative performance of the models. To do so, for each F1, precision, and recall table, we linearly rescale the values in each column so that the maximum value in each column is 1 and the minimum value in each column is 0.

Additional Figures

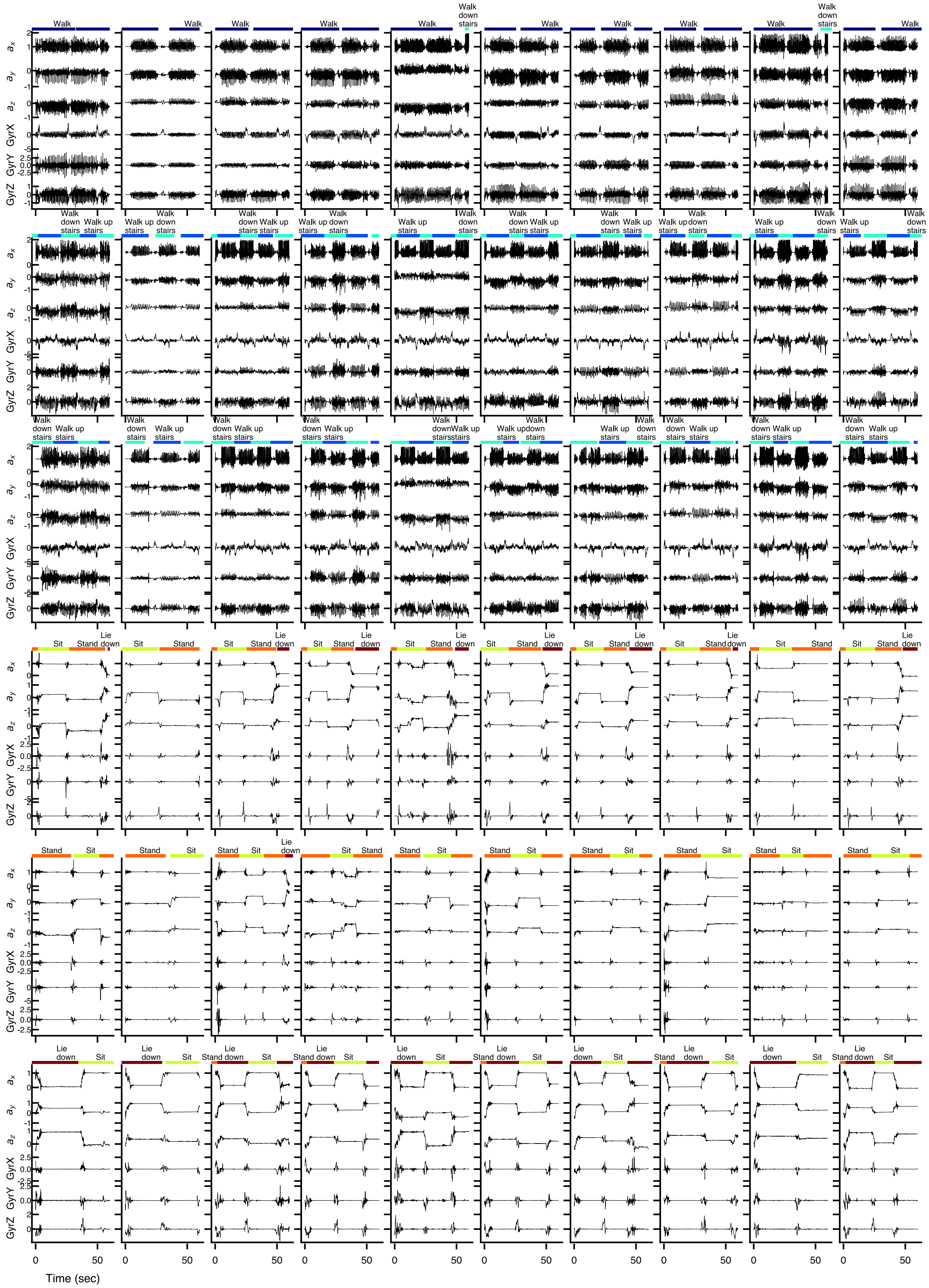

Figure S1: 10 examples of each class in the Human Activity Recognition (HAR) dataset, beginning five seconds before the class onset. Accelerometer units are g, gyroscope units are rad/sec. No information was provided on the axes orientation of the sensors.

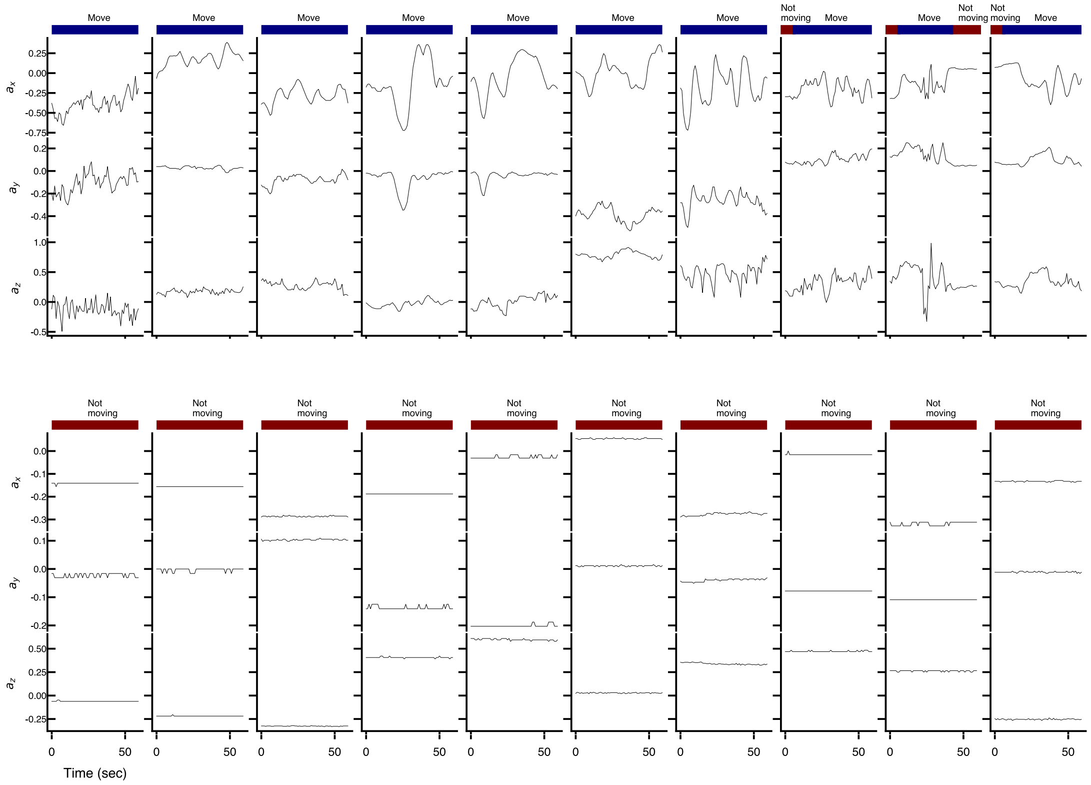

Figure S2: 10 examples of each class in the Rattlesnake dataset, beginning five seconds before the class onset. Accelerometer units are g. Bio-logger was surgically implanted at 2/3 the body length of the rattlesnake. Axes: x (forward- backward), y (left-right), z (up-down).

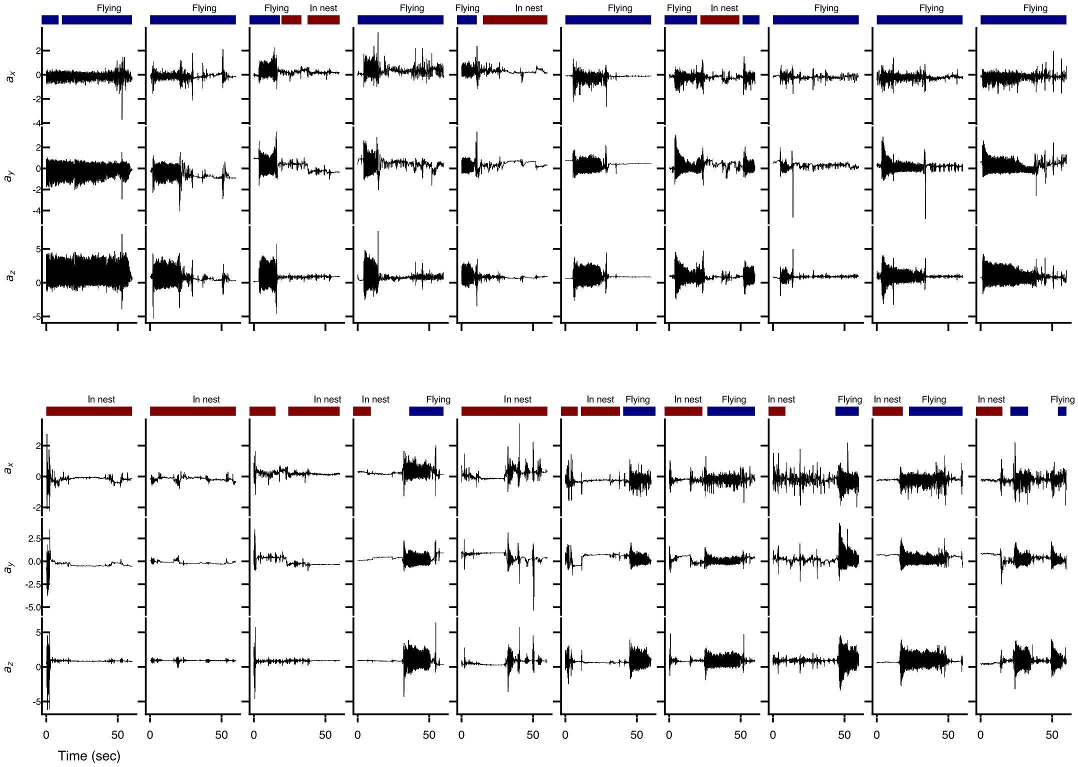

Figure S3: 10 examples of each class in the Crow dataset. Accelerometer units are g. Bio-logger was attached to the base of the crow's tail. Axes: x (backward-forward), y (lateral), z (down-up).

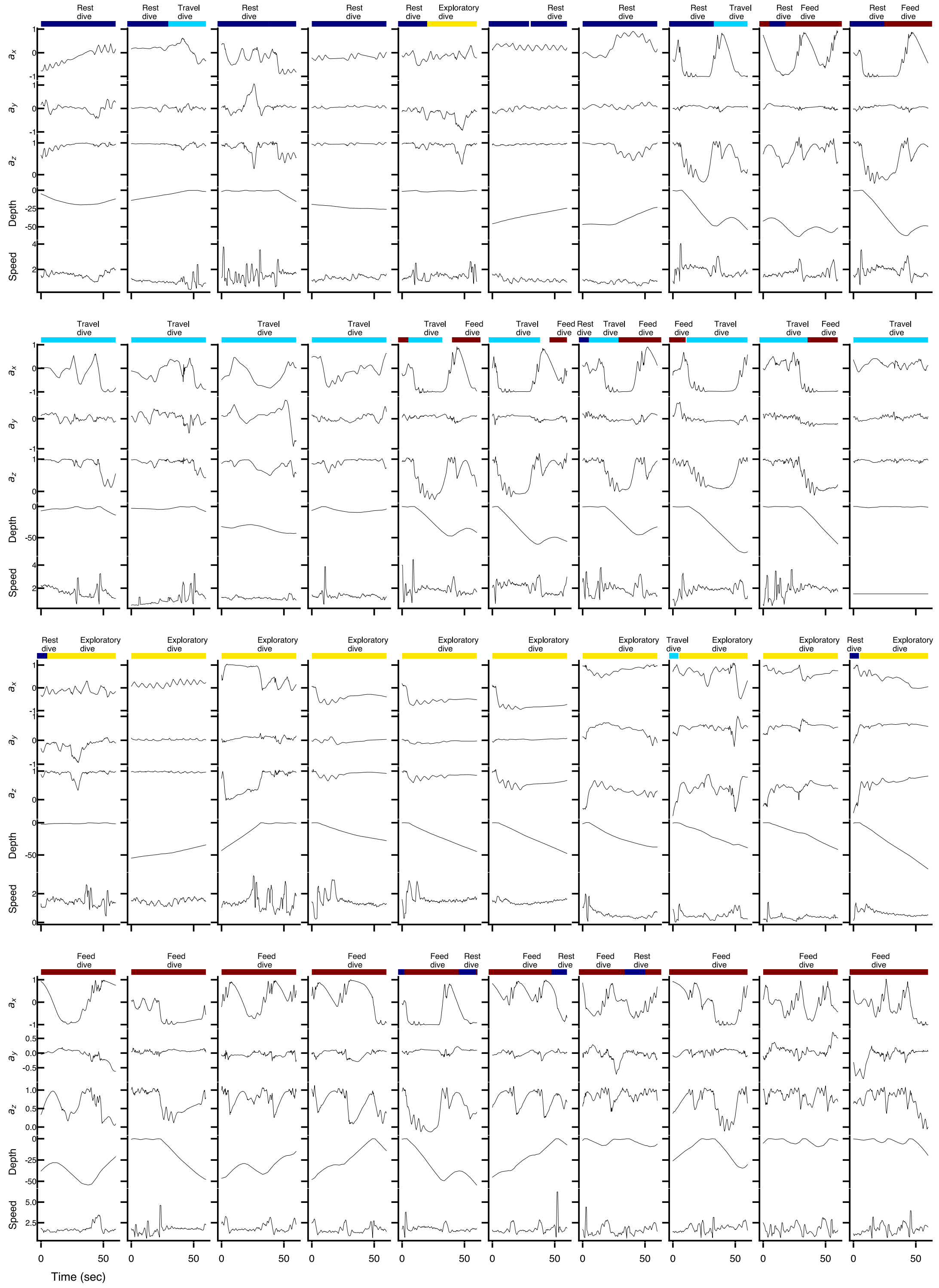

Figure S4: 10 examples of each class in the Whale dataset. Accelerometer units are g. Depth units are m from the water's surface. Speed units are m/s. Bio-loggers were placed on the dorsal surface of the whale or high on the flank, forward of the dorsal fin. Axes: x (backward-forward), y (lateral), z (down-up).

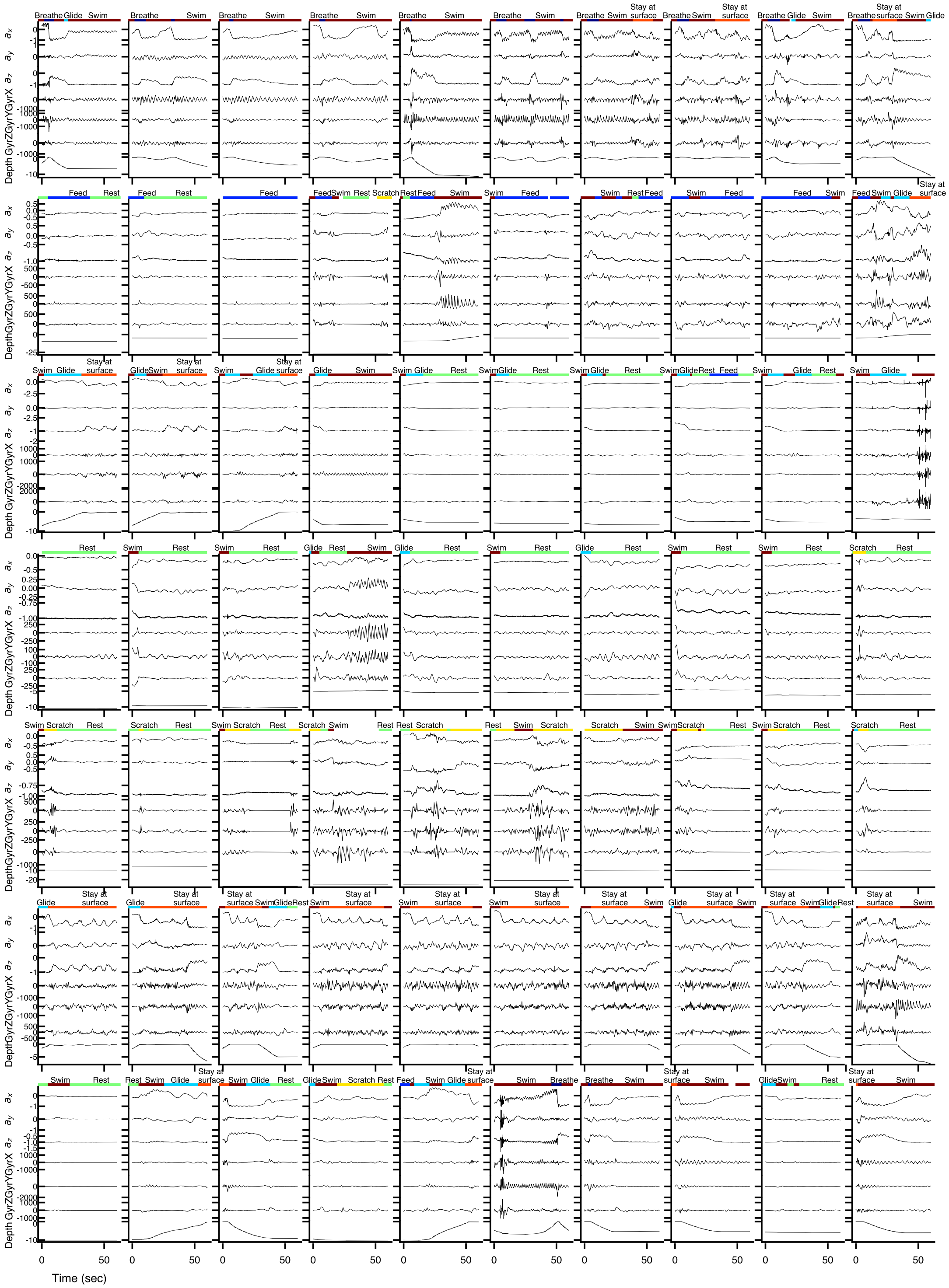

Figure S5: 10 examples of each class in the Turtle dataset. Accelerometer units are g. Gyroscope units are $mrad/sec$. Depth units are m from the water's surface. Bio-loggers were placed on the carapace in a tilted orientation. Axes: x (back-to-front), y (right-to-left), z (bottom-to-top).

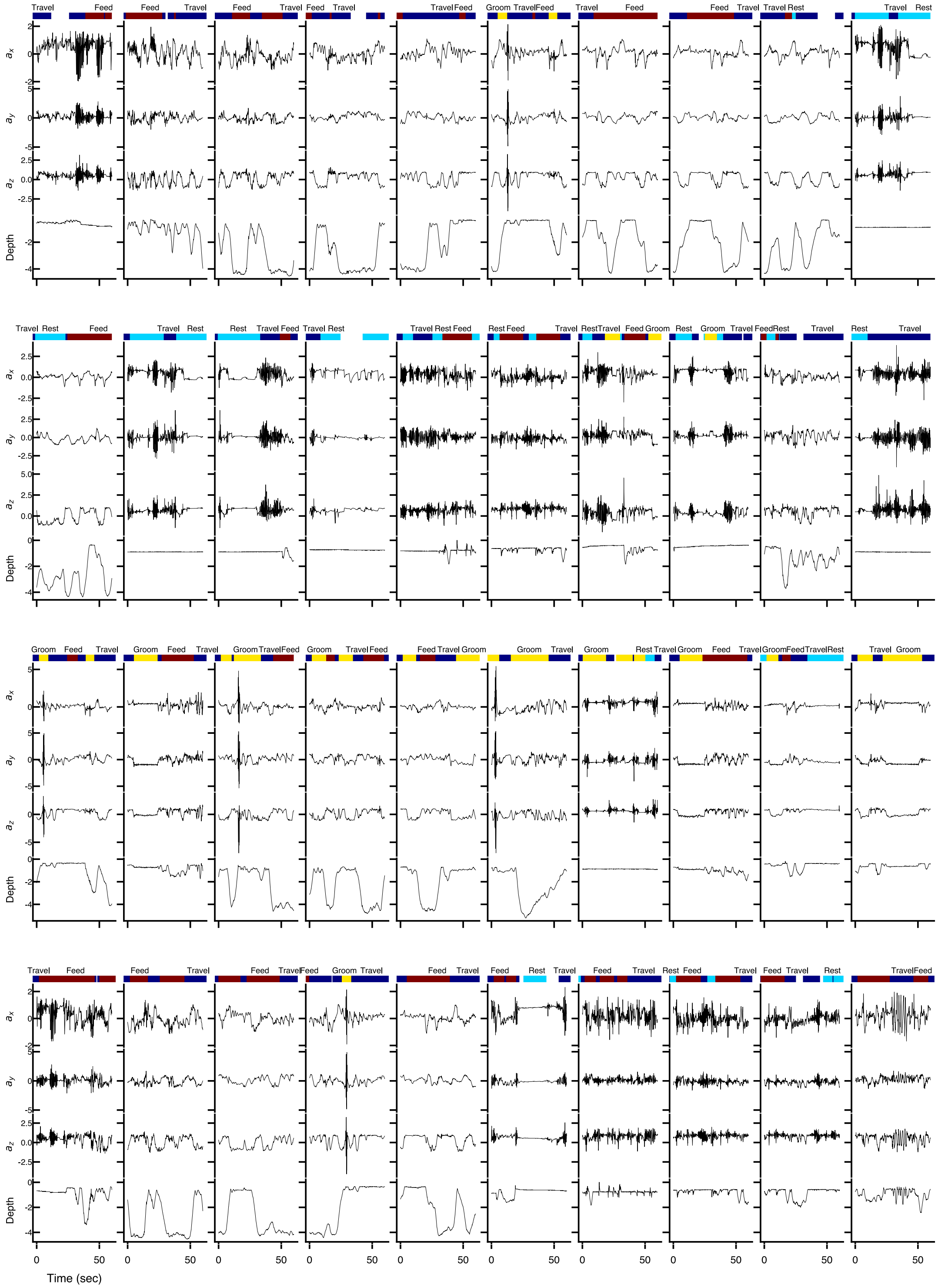

Figure S6: 10 examples of each class in the Seals dataset. Accelerometer units are g. Gyroscope units are $mrad/sec$. Depth units are m from the water's surface. Bio-loggers were placed between the shoulder blades. Axes: x (anterior-posterior), y (lateral), z (dorsal-ventral).

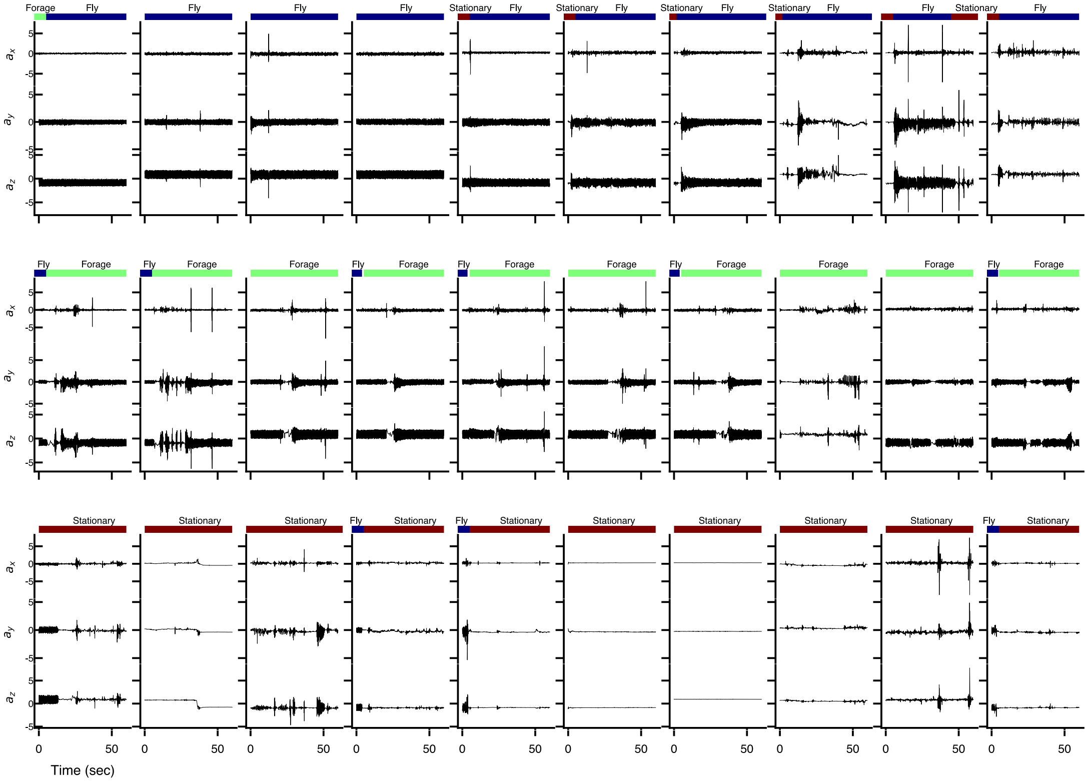

Figure S7: 10 examples of each class in the Gull dataset. Accelerometer units are g. Bio-loggers were placed on the back or abdomen. Axes: x (lateral), y (forward-backward), z (down-up).

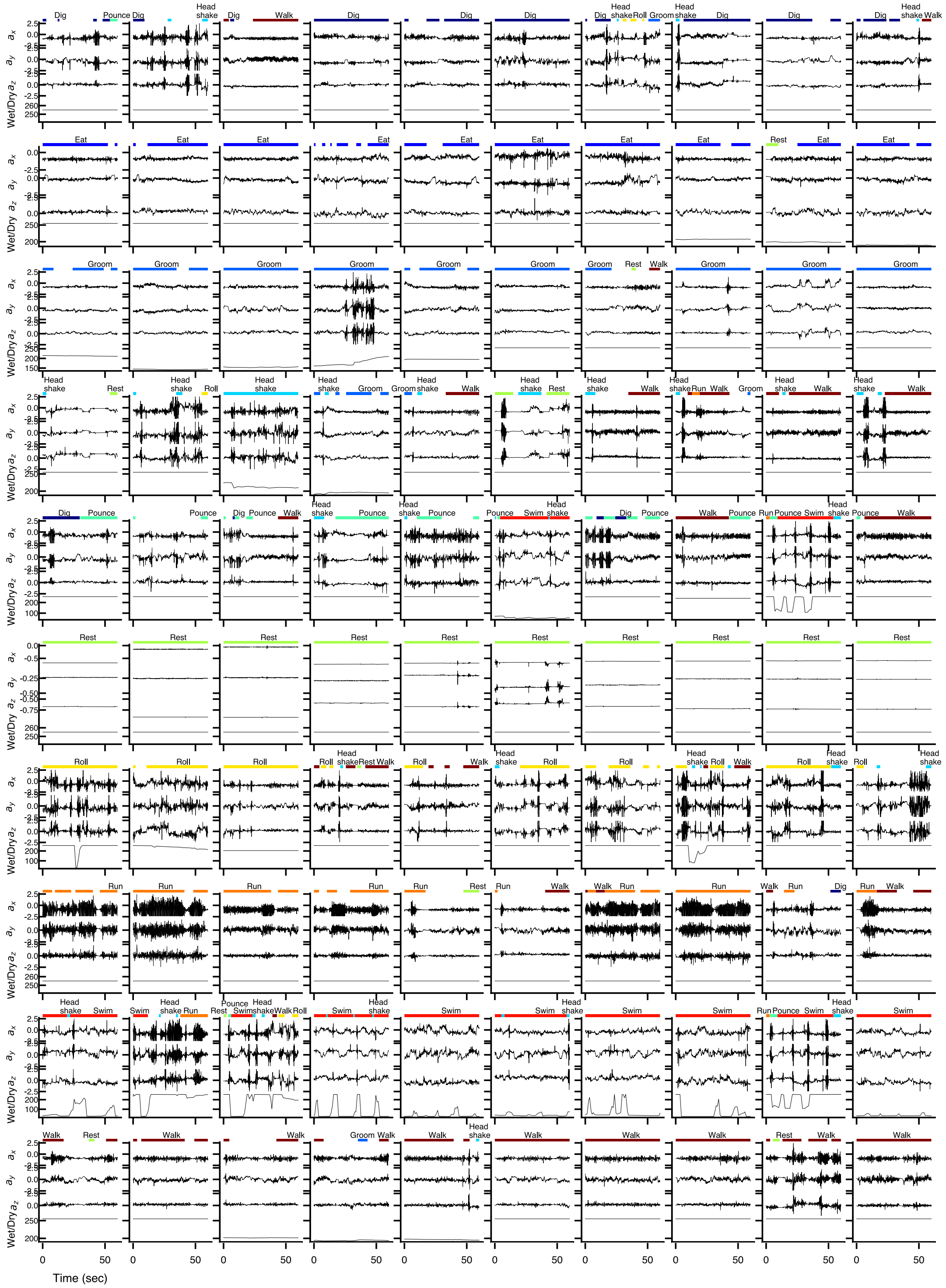

Figure S8: 10 examples of each class in the Polar bear dataset. Accelerometer units are g. The wet sensor ranges from 0(wet)-255(dry). Bio-loggers were placed as a collar around the polar bear's neck. Axes: z (lateral), x (backward-forward), y (down-up).

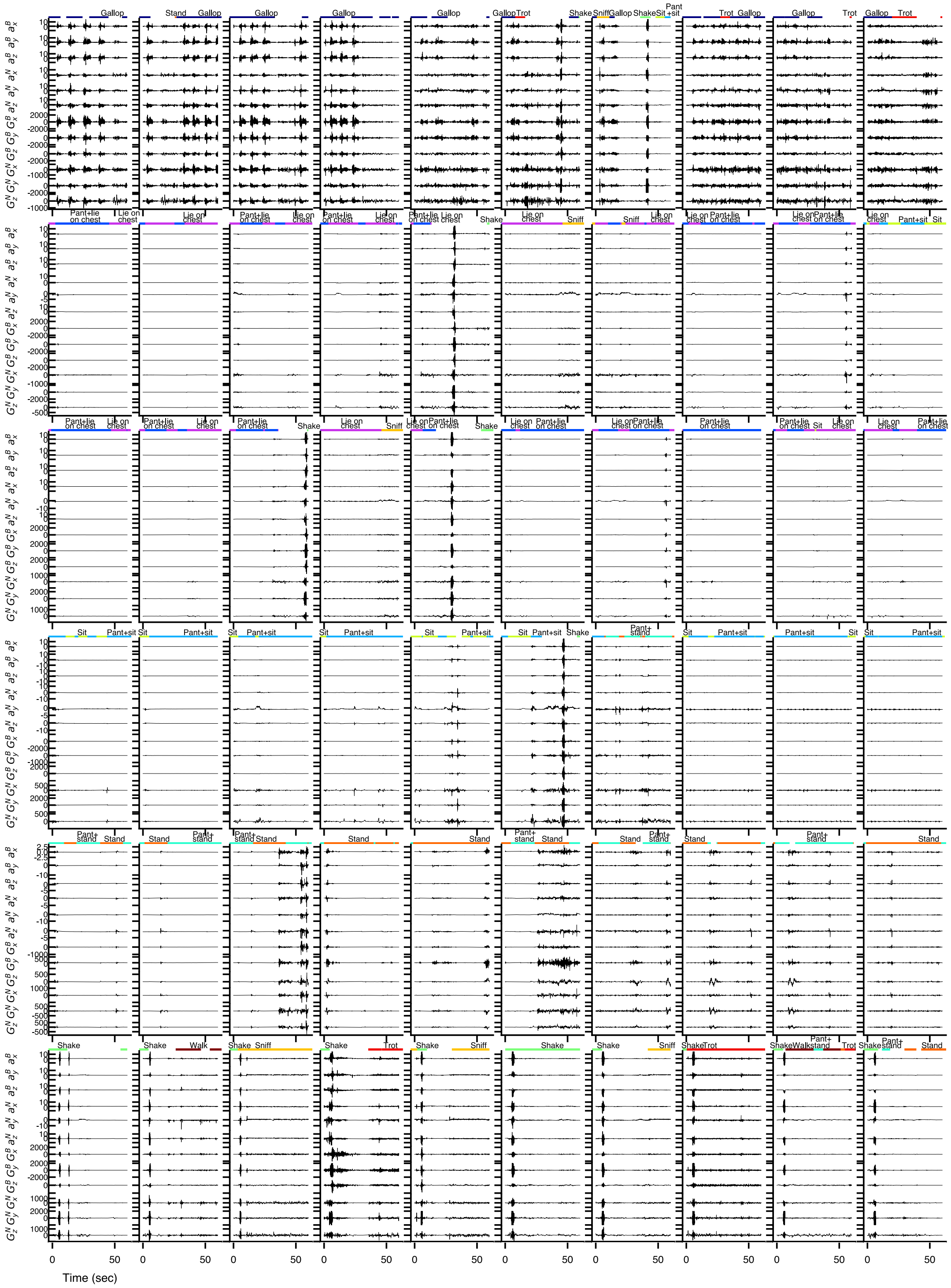

Figure S9: 10 examples of classes in the Dog dataset. See next figure for remaining classes. Accelerometer units are g. No information on axes orientation was provided.

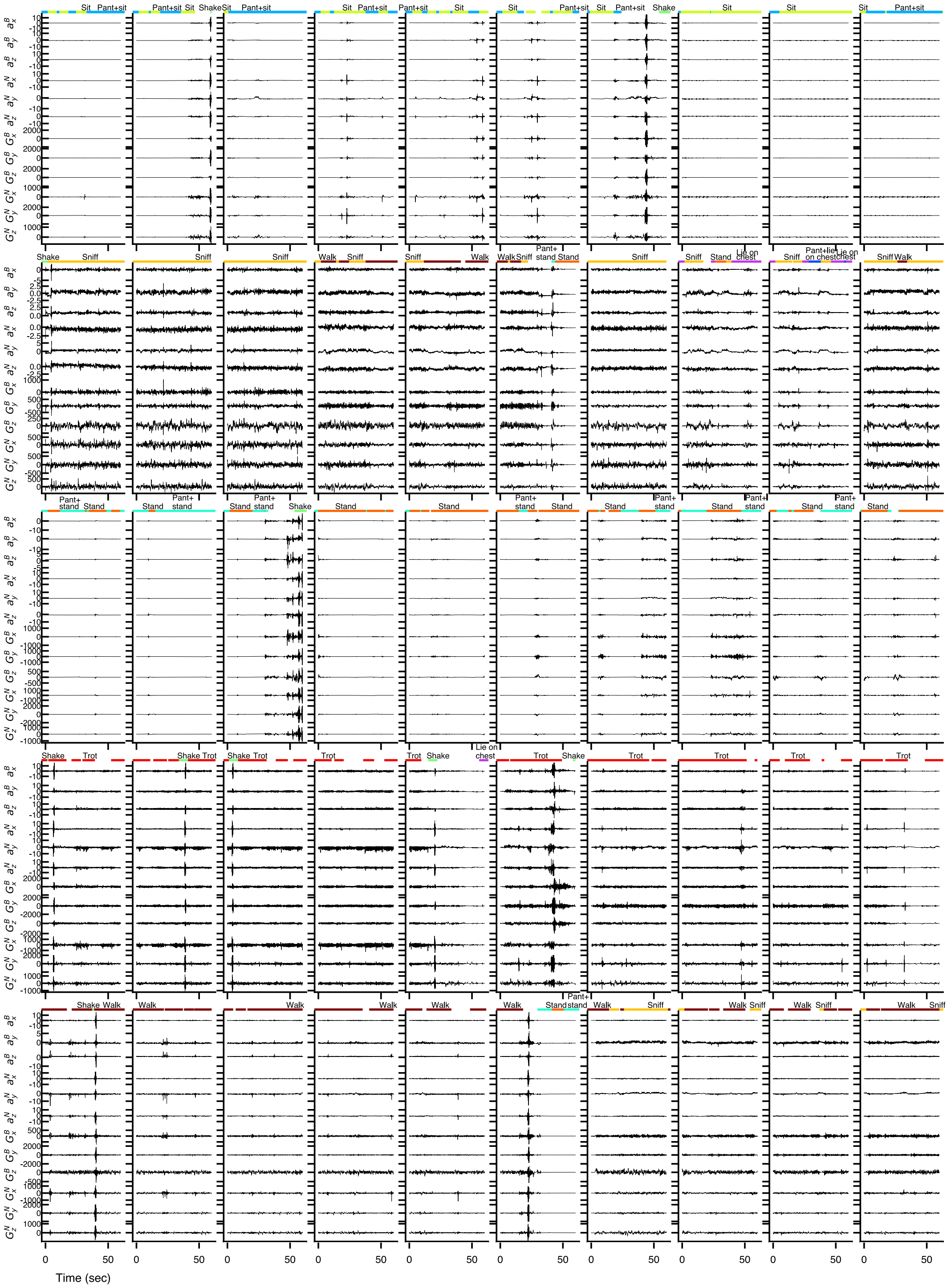

Figure S10: 10 examples of classes in the Dog dataset. See preceding figure for remaining classes. Accelerometer units are g. No information on axes orientation was provided.

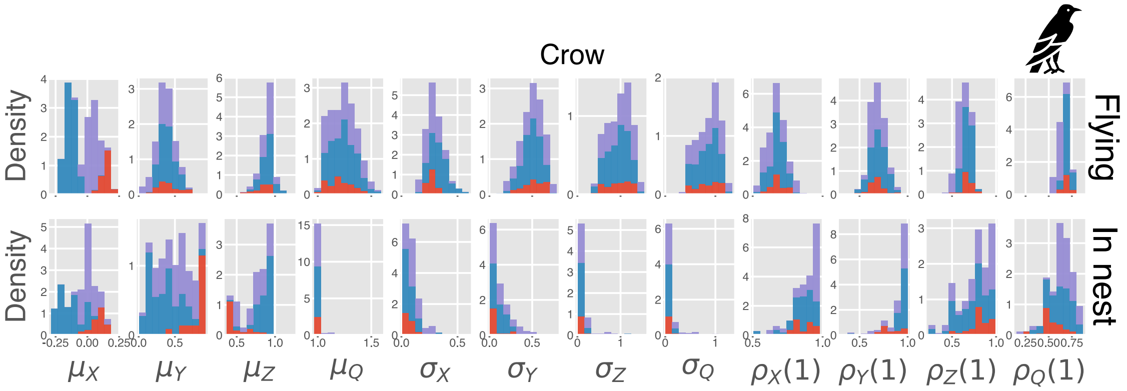

Figure S11: Crow dataset summary statistics of the accelerometer (x, y, z)-axes and q-axis (root-mean-square amplitude), for each class. Each color represents a different individual (not all individuals are shown): the histograms are stacked. μ: mean, σ: standard deviation, $\rho(1)$: one-sample auto-correlation. Summary statistics computed over 20 seconds. These statistics show within and between class variation, as well as between individual variation. For example, note the difference in μ_Q for the two classes.

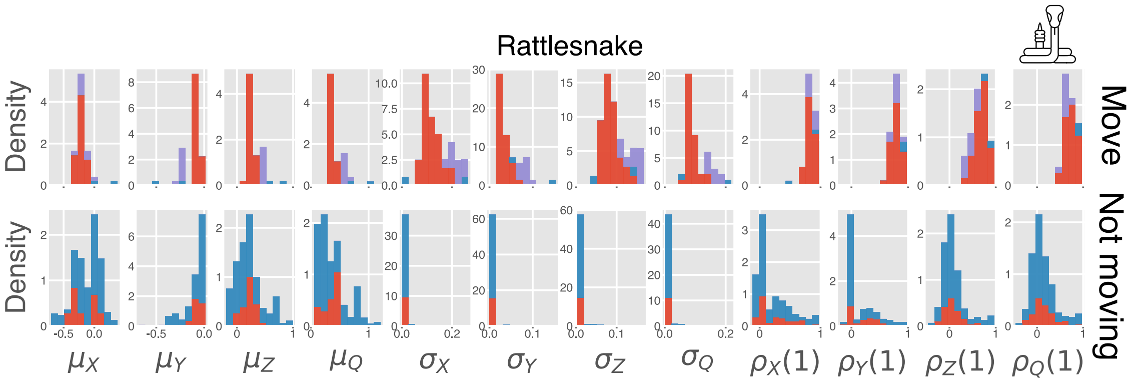

Figure S12: Rattlesnake dataset summary statistics of the accelerometer (x, y, z)-axes and q-axis (root-mean-square amplitude), for each class. Each color represents a different individual (not all individuals are shown): the histograms are stacked. μ: mean, σ: standard deviation, $\rho(1)$: one-sample auto-correlation. Summary statistics computed over 60 seconds. These statistics show within and between class variation, as well as between individual variation. For example, note the difference in the standard deviation statistics between the two classes.

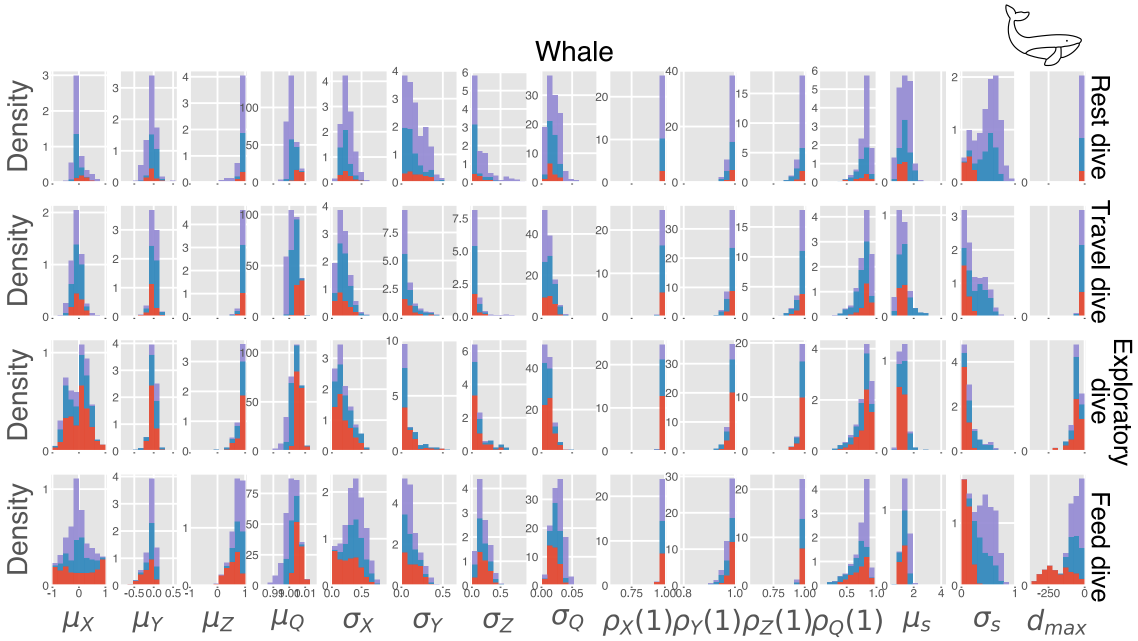

Figure S13: Whale dataset summary statistics of the accelerometer (x, y, z)-axes, q-axis (root-mean-square amplitude), speed s, and depth d for each class. Each color represents a different individual (not all individuals are shown): the histograms are stacked. μ: mean, σ: standard deviation, $\rho(1)$: one-sample auto-correlation. Summary statistics computed over 60 seconds. These statistics show within and between class variation, as well as between individual variation. For example, note the individual variability in the *feeding dives* for μ_X, σ_S, and $d_{\max}$. None of the statistics obviously distinguish the classes.

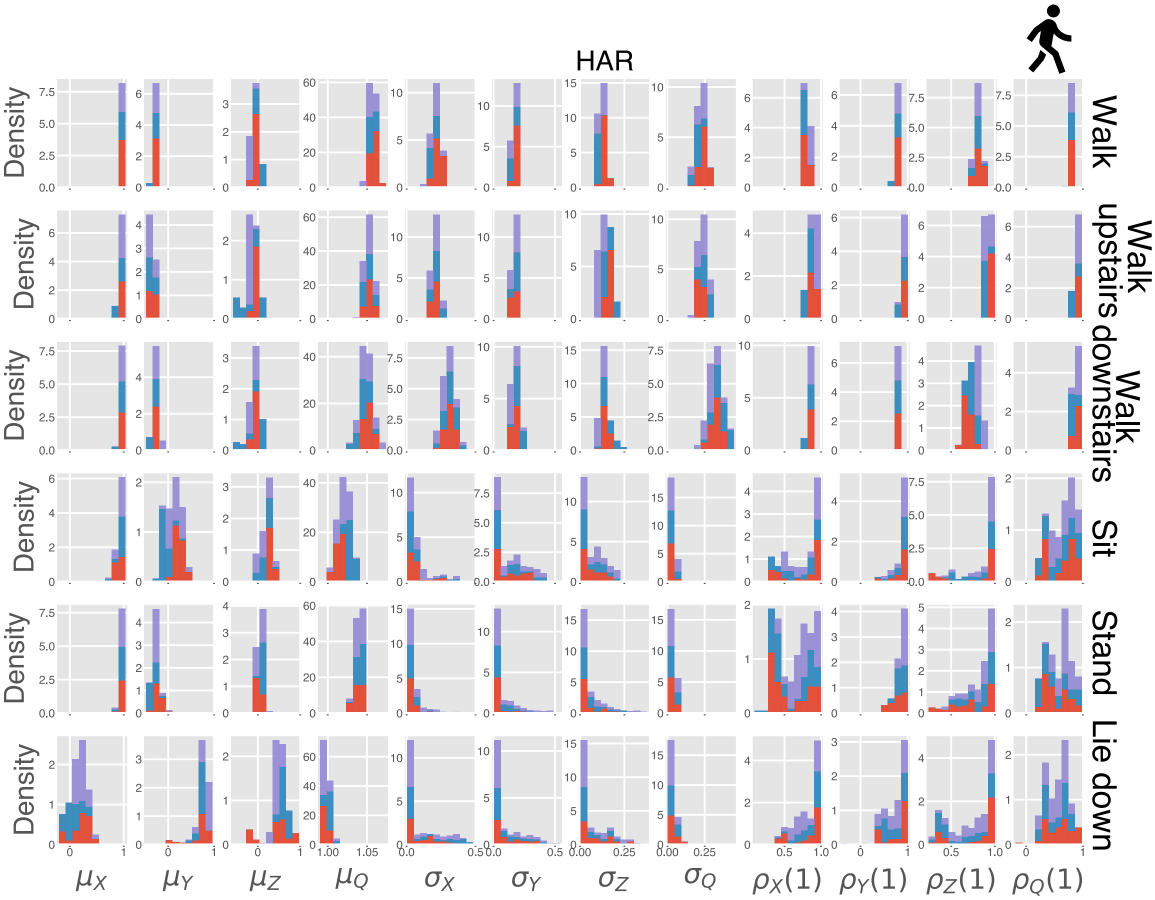

Figure S14: Human dataset summary statistics of the accelerometer (x, y, z)-axes and q-axis (root-mean-square amplitude). Each color represents a different individual (not all individuals are shown): the histograms are stacked. μ: mean, σ: standard deviation, $\rho(1)$: one-sample auto-correlation. Summary statistics computed over 10 seconds. These statistics show within and between class variation, as well as between individual variation. For example, note relatively minimal between individual variability. *Lie down* is highly distinct from other classes based on μ statistics.

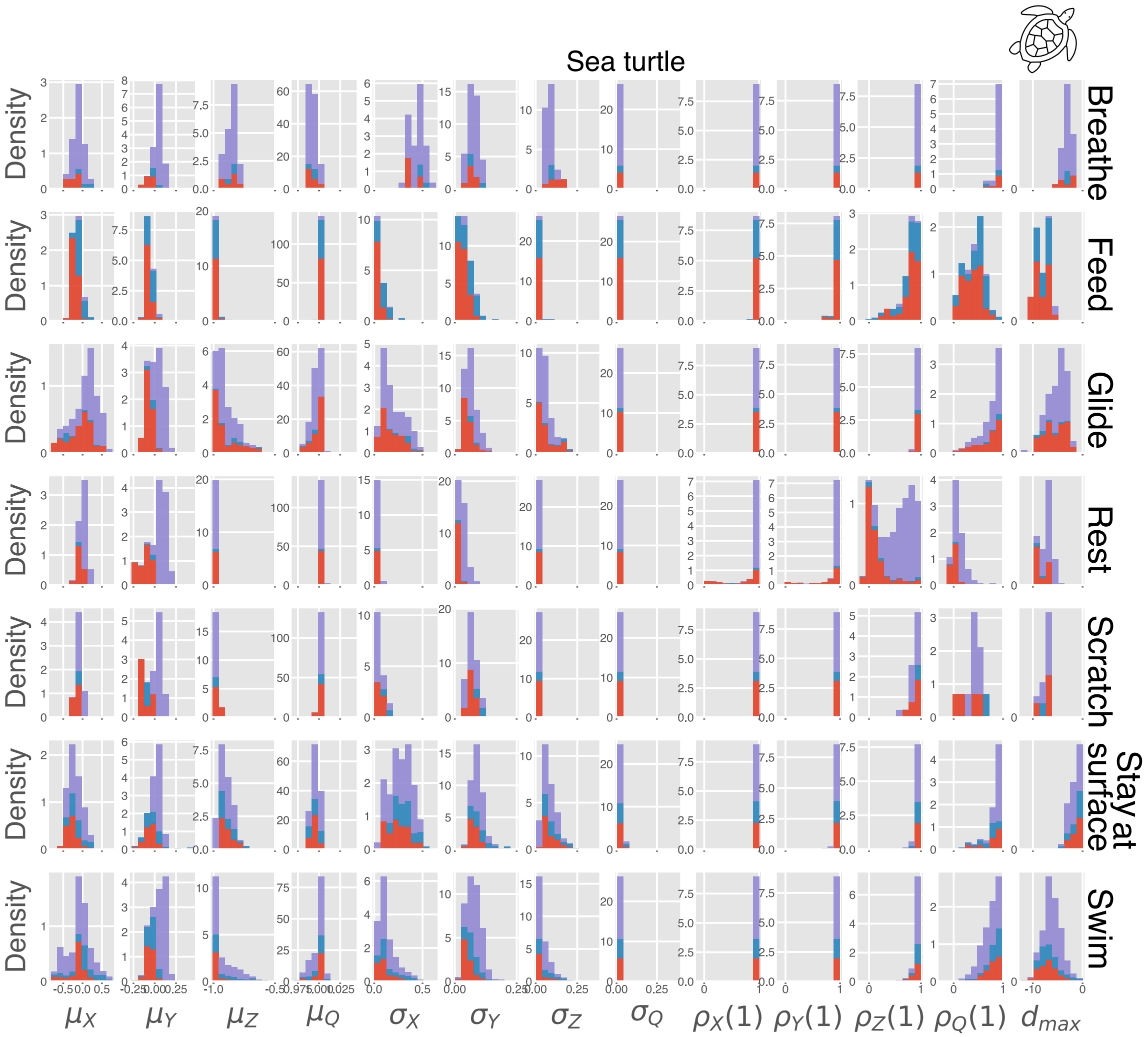

Figure S15: Turtle dataset summary statistics of the accelerometer (x, y, z)-axes and q-axis (root-mean-square amplitude), as well as depth d. Each color represents a different individual (not all individuals are shown): the histograms are stacked. μ: mean, σ: standard deviation, $\rho(1)$: one-sample auto-correlation. Summary statistics computed over 20 seconds. These statistics show within and between class variation, as well as between individual variation. For example, *Stay at surface* and *Breathe* are both characterized by $d_{\max}$ near zero, whereas other behaviors tend to occur at deeper depths.

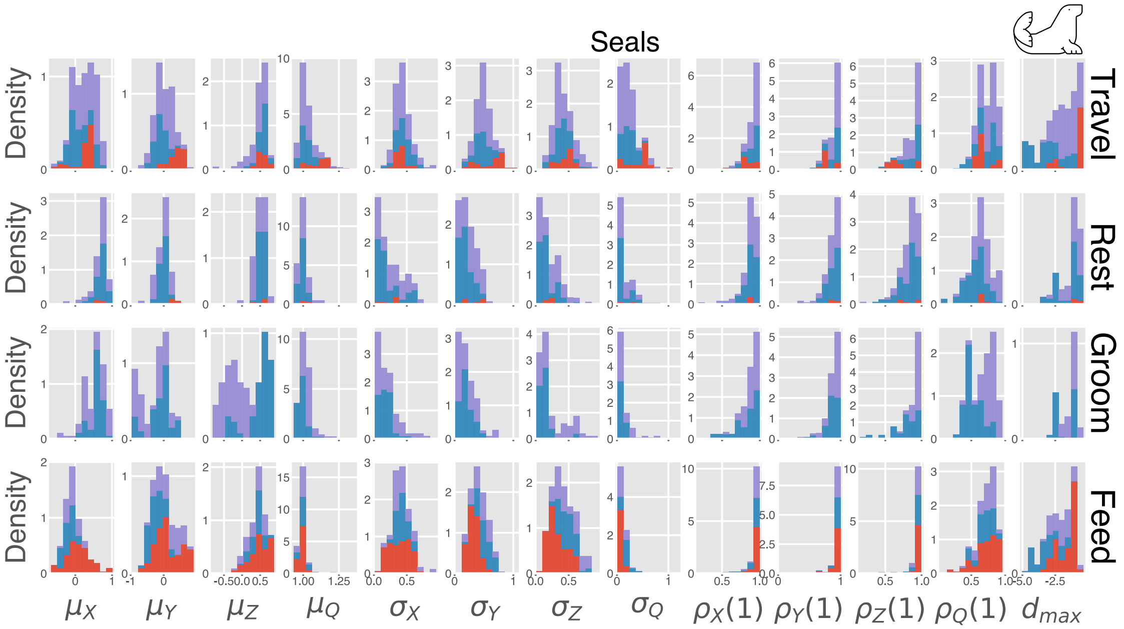

Figure S16: Seals dataset summary statistics of the accelerometer (x, y, z)-axes and q-axis (root-mean-square amplitude), as well as depth d. Each color represents a different individual (not all individuals are shown): the histograms are stacked. μ: mean, σ: standard deviation, $\rho(1)$: one-sample auto-correlation. Summary statistics computed over 10 seconds. These statistics show within and between class variation, as well as between individual variation. For example, `groom` appears to have relatively high between-individual variation, note the bimodality in μ_X and $\rho_Q(1)$.

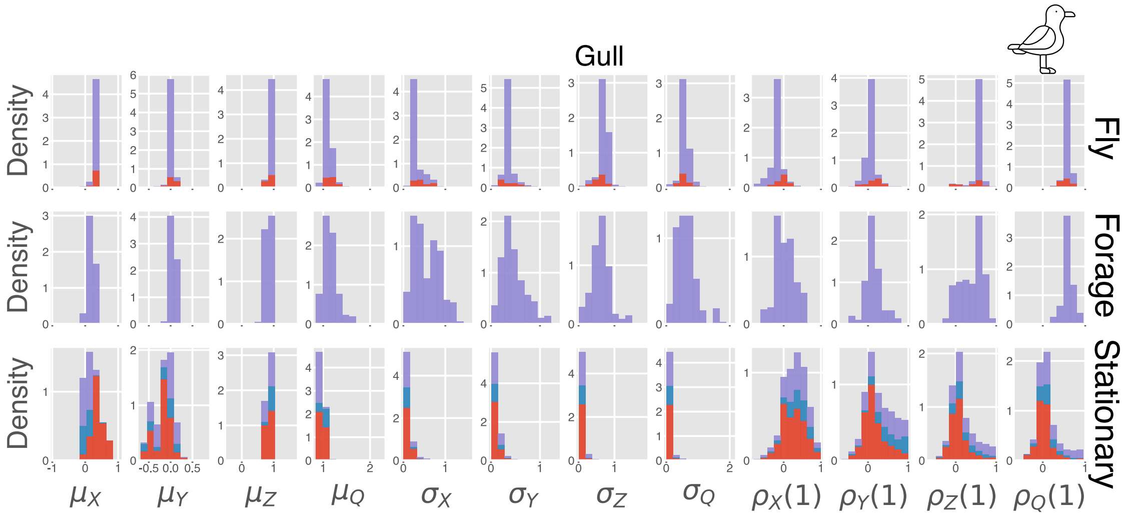

Figure S17: Gull dataset summary statistics of the accelerometer (x, y, z)-axes and q-axis (root-mean-square amplitude). Each color represents a different individual (not all individuals are shown): the histograms are stacked. μ: mean, σ: standard deviation, $\rho(1)$: one-sample auto-correlation. Summary statistics computed over 30 seconds. These statistics show within and between class variation, as well as between individual variation. For example, *stationary* is distinguished from the other classes in the low values of the σ statistics, while *flying* and *foraging* have similar modes. We cannot assess between-individual variability for *foraging* in these statistics: it is a rare class and only one of the three sampled individuals performed this behavior.

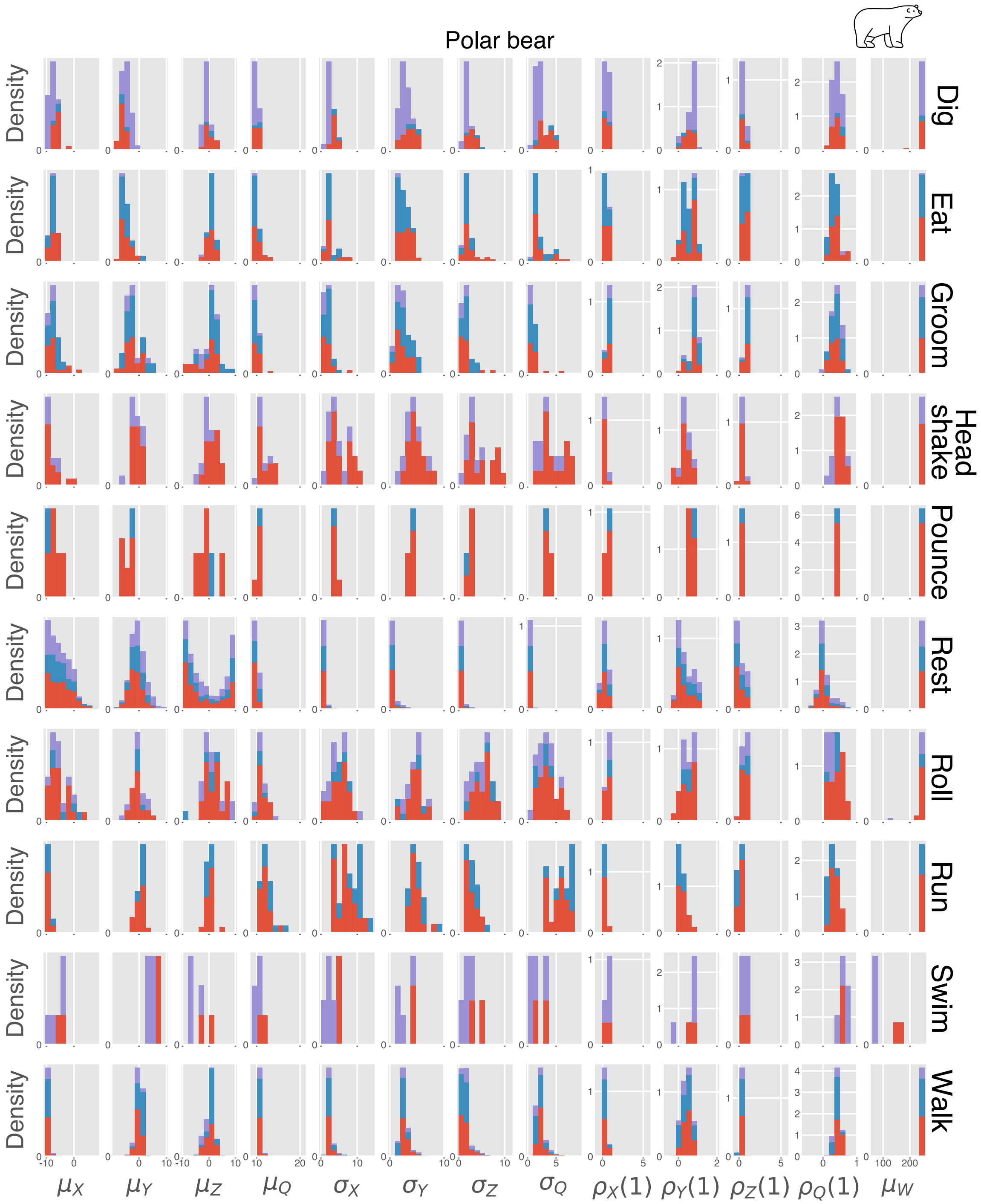

Figure S18: Polar bear dataset summary statistics of the accelerometer (x, y, z)-axes and q-axis (root-mean-square amplitude), as well as the mean wet/dry value. Each color represents a different individual (not all individuals are shown): the histograms are stacked. μ: mean, σ: standard deviation, $\rho(1)$: one-sample auto-correlation. Summary statistics computed over 30 seconds. These statistics show within and between class variation, as well as between individual variation. For example, *head shake* and *roll* have relatively high variation in these summary statistics.

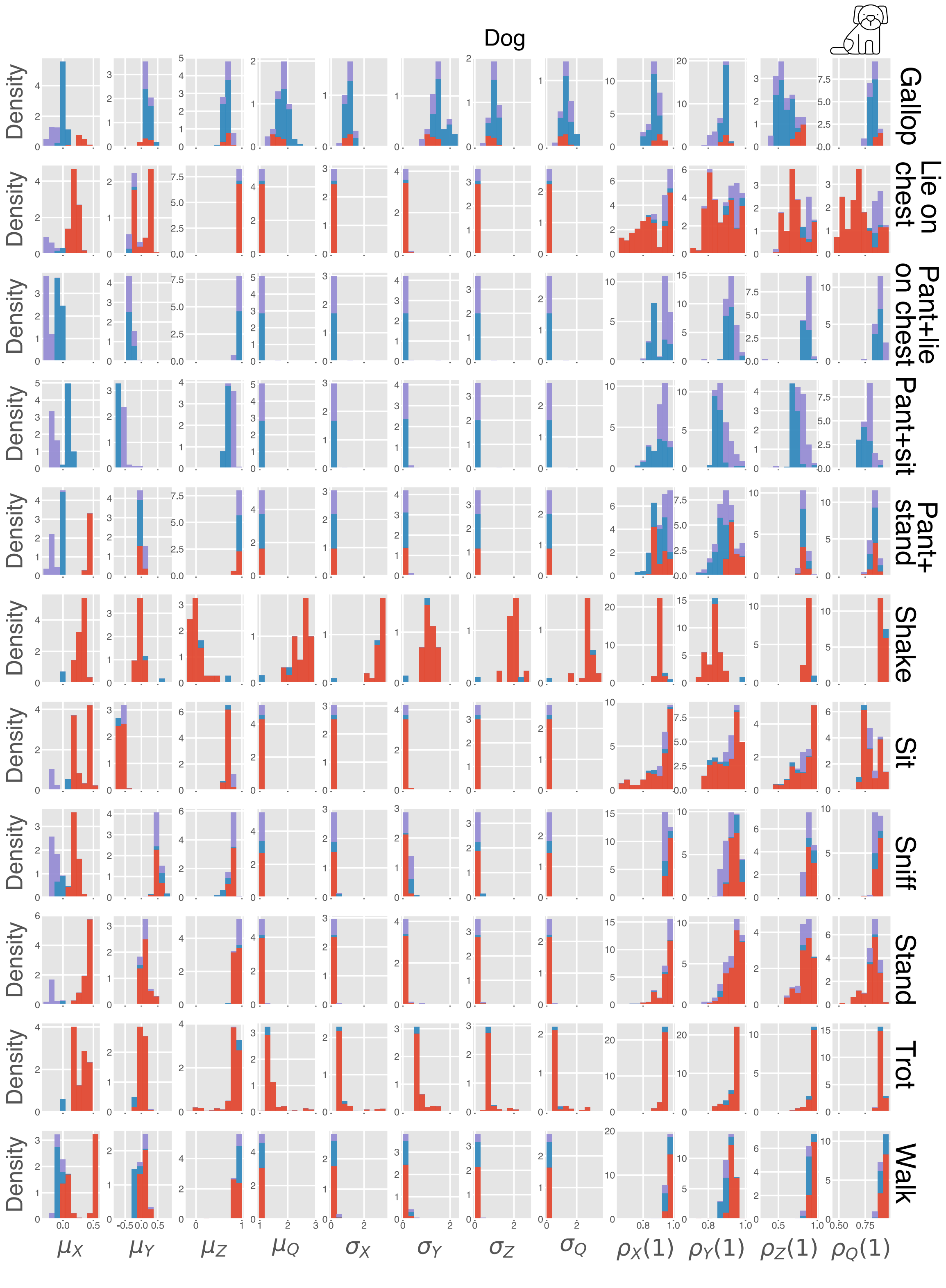

Figure S19: Dog dataset summary statistics of the accelerometer (x, y, z)-axes and q-axis (root-mean-square amplitude), from the sensor placed on the back of the dogs. Each color represents a different individual (not all individuals are shown): the histograms are stacked. μ: mean, σ: standard deviation, $\rho(1)$: one-sample auto-correlation. Summary statistics computed over 30 seconds. These statistics show within and between class variation, as well as between individual variation. For example, one dog (red) panted relatively little. *Trot* appears to be stereotyped behavior, given the summary statistics are highly peaked.

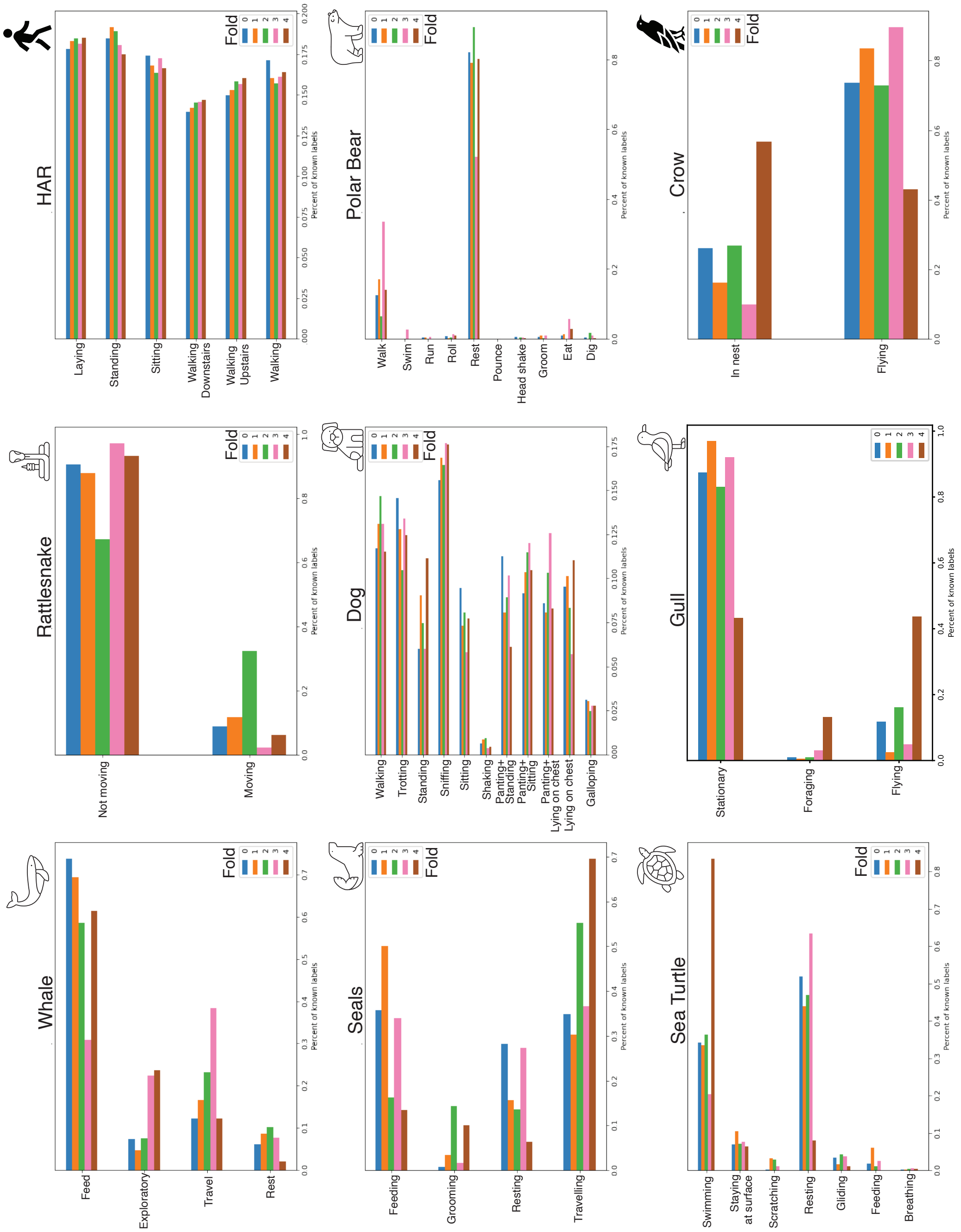

Figure S20: Representation of each behavioral class in the BEBE datasets. The bars represent the proportion of sampled time steps with the given annotation, as a fraction of the total time steps with a known behavioral annotation in that fold. All behavioral classes for each dataset are listed.

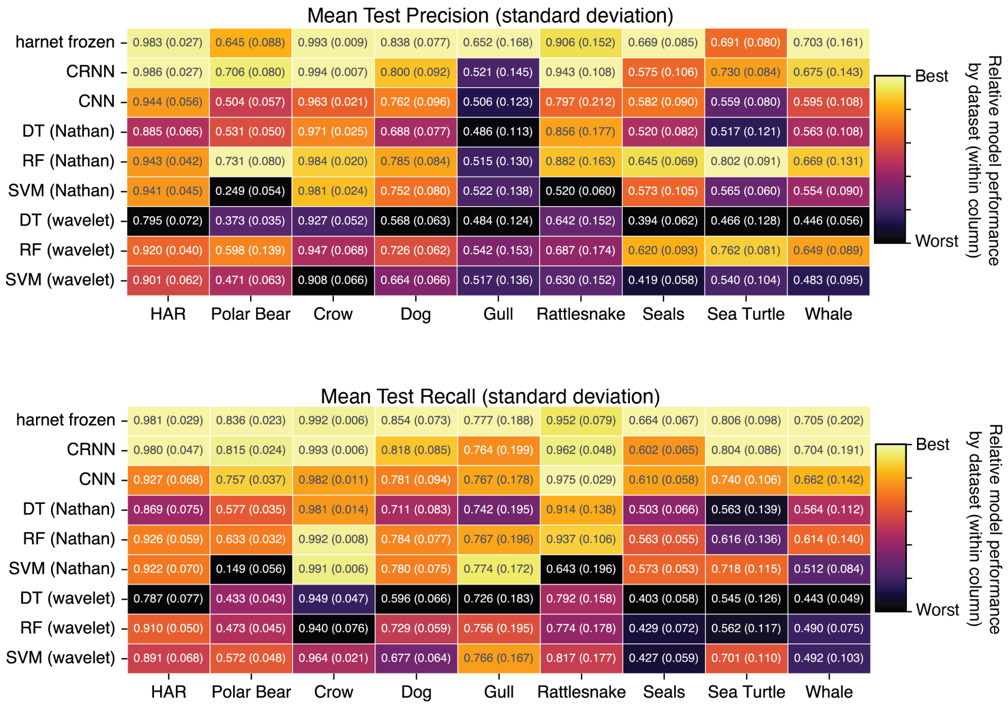

Figure S21: Precision and recall results for deep neural networks and classical models. These scores correspond to Figure 4, using the full data setting and including gyroscope channels. See Figure 4 for color coding. A) Precision. Neural networks (`harnet frozen` and `CRNN`) perform best on 7/9 datasets, while `RF (Nathan` performs best on two datasets. B) Recall. One of the three neural networks shows the best performance across all nine datasets.

A Mean Test Precision: gyroscope omitted, full data

	HAR	Polar Bear	Crow	Dog	Gull	Rattlesnake	Seals	Sea Turtle	Whale
harnet frozen	0.983 (0.027)	0.645 (0.088)	0.993 (0.009)	0.838 (0.077)	0.652 (0.168)	0.906 (0.152)	0.669 (0.085)	0.691 (0.080)	0.703 (0.161)
harnet unfrozen	0.753 (0.048)	0.361 (0.023)	0.865 (0.073)	0.366 (0.067)	0.424 (0.126)	0.846 (0.175)	0.421 (0.041)	0.523 (0.066)	0.710 (0.125)
harnet random	0.569 (0.058)	0.161 (0.036)	0.859 (0.073)	0.252 (0.053)	0.338 (0.020)	0.559 (0.097)	0.398 (0.045)	0.452 (0.076)	0.574 (0.076)
RNN	0.954 (0.062)	0.606 (0.071)	0.980 (0.017)	0.679 (0.097)	0.537 (0.117)	0.876 (0.173)	0.578 (0.086)	0.482 (0.048)	0.634 (0.135)
CRNN	0.985 (0.027)	0.706 (0.080)	0.994 (0.007)	0.779 (0.096)	0.521 (0.145)	0.943 (0.108)	0.575 (0.106)	0.670 (0.083)	0.675 (0.143)
RF (Nathan)	0.918 (0.047)	0.731 (0.080)	0.984 (0.020)	0.765 (0.089)	0.515 (0.130)	0.882 (0.163)	0.645 (0.069)	0.769 (0.086)	0.669 (0.131)

B Mean Test Recall: gyroscope omitted, full data

	HAR	Polar Bear	Crow	Dog	Gull	Rattlesnake	Seals	Sea Turtle	Whale
harnet frozen	0.981 (0.029)	0.836 (0.023)	0.992 (0.006)	0.854 (0.073)	0.777 (0.188)	0.952 (0.079)	0.664 (0.067)	0.806 (0.098)	0.705 (0.202)
harnet unfrozen	0.736 (0.051)	0.326 (0.052)	0.778 (0.085)	0.426 (0.067)	0.594 (0.268)	0.600 (0.200)	0.365 (0.036)	0.667 (0.115)	0.735 (0.149)
harnet random	0.586 (0.058)	0.292 (0.036)	0.714 (0.079)	0.332 (0.064)	0.585 (0.259)	0.680 (0.184)	0.340 (0.044)	0.553 (0.160)	0.597 (0.113)
RNN	0.943 (0.076)	0.796 (0.047)	0.981 (0.020)	0.700 (0.092)	0.761 (0.199)	0.866 (0.174)	0.588 (0.058)	0.683 (0.109)	0.659 (0.202)
CRNN	0.979 (0.041)	0.815 (0.024)	0.993 (0.006)	0.798 (0.092)	0.764 (0.199)	0.962 (0.048)	0.602 (0.065)	0.818 (0.092)	0.704 (0.191)
RF (Nathan)	0.886 (0.070)	0.633 (0.032)	0.992 (0.008)	0.779 (0.085)	0.767 (0.196)	0.937 (0.106)	0.563 (0.055)	0.597 (0.147)	0.614 (0.140)

C Mean Test Precision: gyroscope omitted, reduced data

	HAR	Polar Bear	Crow	Dog	Gull	Rattlesnake	Seals	Sea Turtle	Whale
harnet frozen	0.948 (0.074)	0.586 (0.075)	0.988 (0.012)	0.784 (0.093)	0.560 (0.133)	0.864 (0.159)	0.597 (0.083)	0.662 (0.081)	0.673 (0.100)
harnet unfrozen	0.667 (0.051)	0.337 (0.107)	0.804 (0.064)	0.311 (0.063)	0.515 (0.146)	0.749 (0.201)	0.384 (0.065)	0.467 (0.048)	0.574 (0.076)
harnet random	0.519 (0.078)	0.208 (0.025)	0.845 (0.085)	0.250 (0.059)	0.381 (0.128)	0.715 (0.218)	0.410 (0.115)	0.398 (0.030)	0.530 (0.069)
RNN	0.933 (0.085)	0.501 (0.053)	0.973 (0.028)	0.639 (0.084)	0.452 (0.105)	0.560 (0.157)	0.535 (0.107)	0.657 (0.234)	0.521 (0.140)
RNN wavelet	0.921 (0.065)	0.548 (0.111)	0.964 (0.050)	0.654 (0.072)	0.481 (0.120)	0.822 (0.186)	0.486 (0.131)	0.604 (0.102)	0.457 (0.086)
CRNN	0.942 (0.093)	0.584 (0.071)	0.895 (0.132)	0.657 (0.073)	0.554 (0.151)	0.747 (0.211)	0.553 (0.105)	0.658 (0.100)	0.602 (0.115)
RF (Nathan)	0.884 (0.064)	0.676 (0.094)	0.936 (0.086)	0.720 (0.099)	0.563 (0.122)	0.814 (0.188)	0.616 (0.093)	0.801 (0.080)	0.656 (0.115)

D Recall (Test set): gyroscope omitted, reduced data

	HAR	Polar Bear	Crow	Dog	Gull	Rattlesnake	Seals	Sea Turtle	Whale
harnet frozen	0.941 (0.077)	0.724 (0.110)	0.984 (0.015)	0.791 (0.092)	0.802 (0.173)	0.942 (0.144)	0.603 (0.071)	0.770 (0.102)	0.632 (0.146)
harnet unfrozen	0.650 (0.051)	0.295 (0.093)	0.662 (0.082)	0.380 (0.067)	0.585 (0.275)	0.609 (0.196)	0.353 (0.044)	0.605 (0.134)	0.611 (0.106)
harnet random	0.517 (0.072)	0.307 (0.050)	0.663 (0.060)	0.313 (0.055)	0.605 (0.252)	0.609 (0.187)	0.307 (0.037)	0.521 (0.160)	0.547 (0.085)
RNN	0.922 (0.081)	0.661 (0.121)	0.976 (0.025)	0.650 (0.081)	0.753 (0.176)	0.623 (0.235)	0.492 (0.073)	0.397 (0.291)	0.510 (0.187)
RNN wavelet	0.904 (0.089)	0.661 (0.129)	0.966 (0.053)	0.671 (0.070)	0.758 (0.185)	0.764 (0.225)	0.488 (0.077)	0.727 (0.152)	0.466 (0.096)
CRNN	0.930 (0.100)	0.663 (0.132)	0.870 (0.179)	0.652 (0.085)	0.738 (0.221)	0.820 (0.226)	0.523 (0.080)	0.714 (0.138)	0.608 (0.187)
RF (Nathan)	0.843 (0.081)	0.537 (0.096)	0.923 (0.117)	0.722 (0.093)	0.743 (0.222)	0.925 (0.121)	0.438 (0.098)	0.503 (0.158)	0.528 (0.150)

E Difference in Test Precision: gyroscope omitted, reduced data - full data

	HAR	Polar Bear	Crow	Dog	Gull	Rattlesnake	Seals	Sea Turtle	Whale
harnet frozen	-0.036 (0.059)	-0.059 (0.025)	-0.005 (0.008)	-0.054 (0.050)	-0.092 (0.146)	-0.042 (0.098)	-0.073 (0.075)	-0.029 (0.021)	-0.030 (0.133)
CRNN	-0.043 (0.082)	-0.121 (0.024)	-0.099 (0.126)	-0.123 (0.057)	0.032 (0.176)	-0.196 (0.218)	-0.022 (0.050)	-0.012 (0.072)	-0.073 (0.093)
RF (Nathan)	-0.034 (0.063)	-0.056 (0.041)	-0.049 (0.086)	-0.046 (0.053)	0.048 (0.115)	-0.069 (0.275)	-0.029 (0.105)	0.032 (0.063)	-0.014 (0.115)

F Difference in Test Recall: gyroscope omitted, reduced data - full data

	HAR	Polar Bear	Crow	Dog	Gull	Rattlesnake	Seals	Sea Turtle	Whale
harnet frozen	-0.040 (0.057)	-0.111 (0.090)	-0.008 (0.011)	-0.063 (0.051)	0.025 (0.038)	-0.010 (0.134)	-0.061 (0.056)	-0.037 (0.037)	-0.073 (0.145)
CRNN	-0.050 (0.079)	-0.152 (0.113)	-0.124 (0.178)	-0.146 (0.064)	-0.027 (0.075)	-0.142 (0.247)	-0.079 (0.041)	-0.105 (0.070)	-0.096 (0.143)
RF (Nathan)	-0.043 (0.098)	-0.095 (0.083)	-0.070 (0.115)	-0.057 (0.057)	-0.024 (0.069)	-0.012 (0.114)	-0.126 (0.093)	-0.094 (0.037)	-0.086 (0.116)

Relative model performance by dataset (within column): Best – Worst

Figure S22: Precision and recall results for experiments without gyroscope in both the full data and reduced data settings. Panels A/B correspond to Figure 5D, panels C/D correspond to Figure 5E, and E/F correspond to Figure 5F. See Figure 4 for color coding. A) Precision for full data experiments with gyroscope omitted. `harnet frozen` performs best on 3/9 datasets, and `CRNN` performs best on 3/9 datsets. B) Recall for full data experiments with gyroscope omitted. `harnet frozen` performs best on 5/9 datasets. C) Precision for reduced data experiments with gyroscope omitted. `harnet frozen` performs best on 5/9 datasets. D) Recall for reduced data experiments with gyroscope omitted. `harnet frozen` performs best on all datasets. E) Difference in precision scores for reduced data and full data setting. `RF` shows the smallest decrease in performance in the reduced data setting, across 5/9 datasets. F) Difference in recall scores for reduced data and full data setting. `harnet frozen` shows the smallest decrease in performance in the reduced data setting on 6/9 datasets.

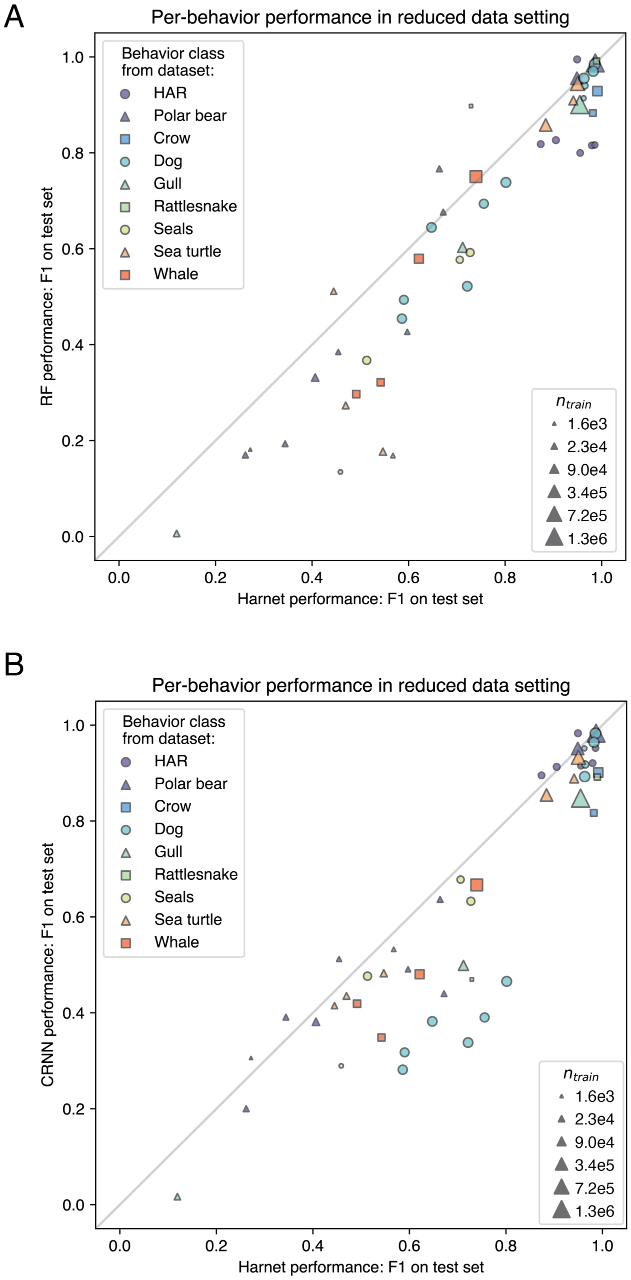

Figure S23: Performance of `harnet` compared to A) `RF` and B) `CRNN` in the reduced data setting, by behavior class. `harnet` performance is better than the other models on most behavior classes, including classes with both relatively small and large train set sizes.

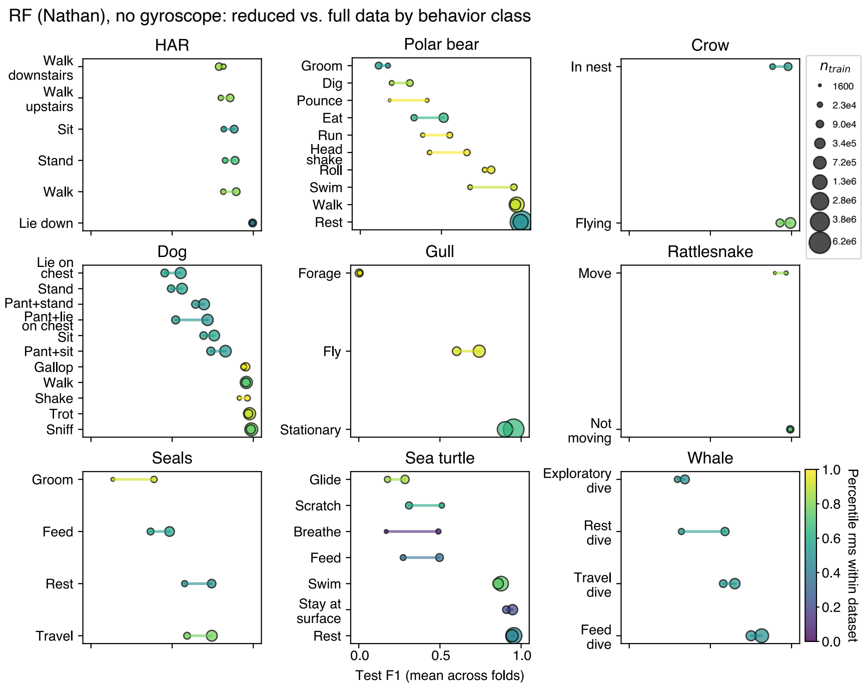

Figure S24: Performance of `RF (Nathan)` in reduced and full data settings, by behavior class. Size of the marker indicates training dataset size (mean across folds). Color indicates the percentile of the average root-mean-square amplitude of datapoints in that class, as compared to the root-mean-square (rms) amplitude of all labeled datapoints.

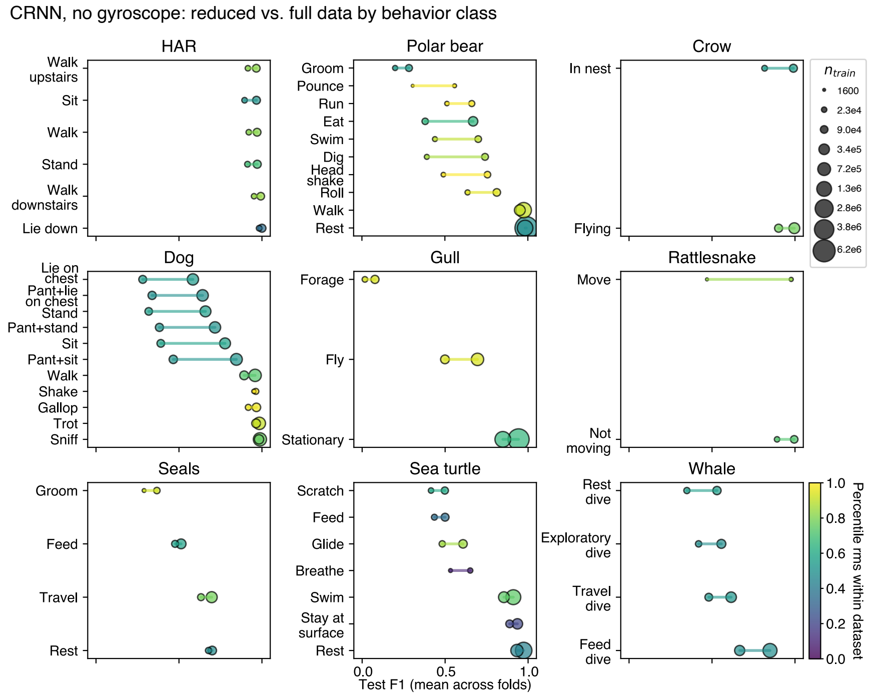

Figure S25: Performance of `CRNN` in reduced and full data settings, by behavior class. See previous caption.

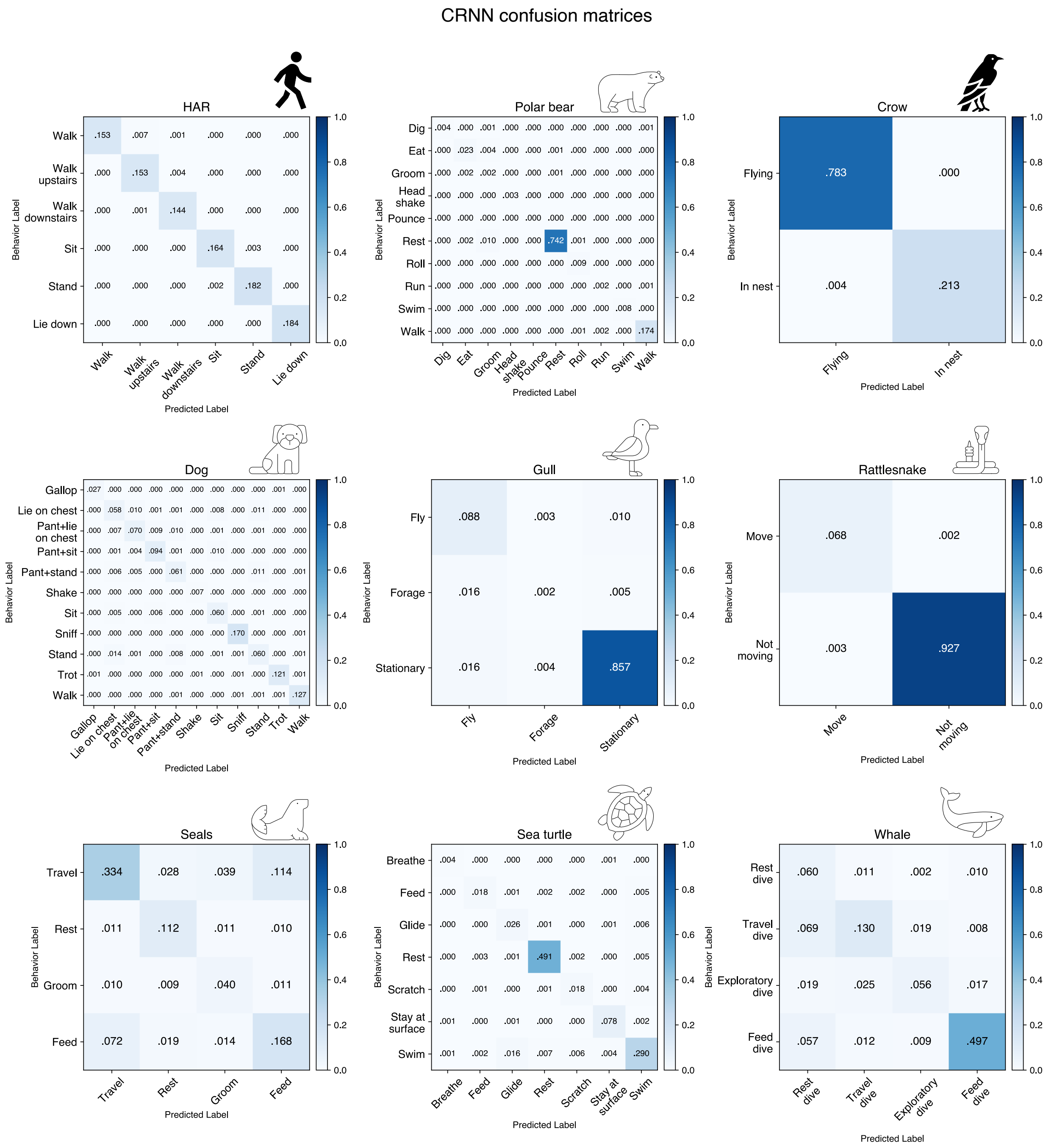

Figure S26: Confusion matrices for CRNN predictions versus behavioral labels, for all nine datasets in BEBE. Numbers represent the fraction of total labeled data. Computed for data taken from the test sets of the four cross validation steps that were not used for hyperparameter selection. Confusion matrices for the other experiments can be found on the Zenodo data repository.

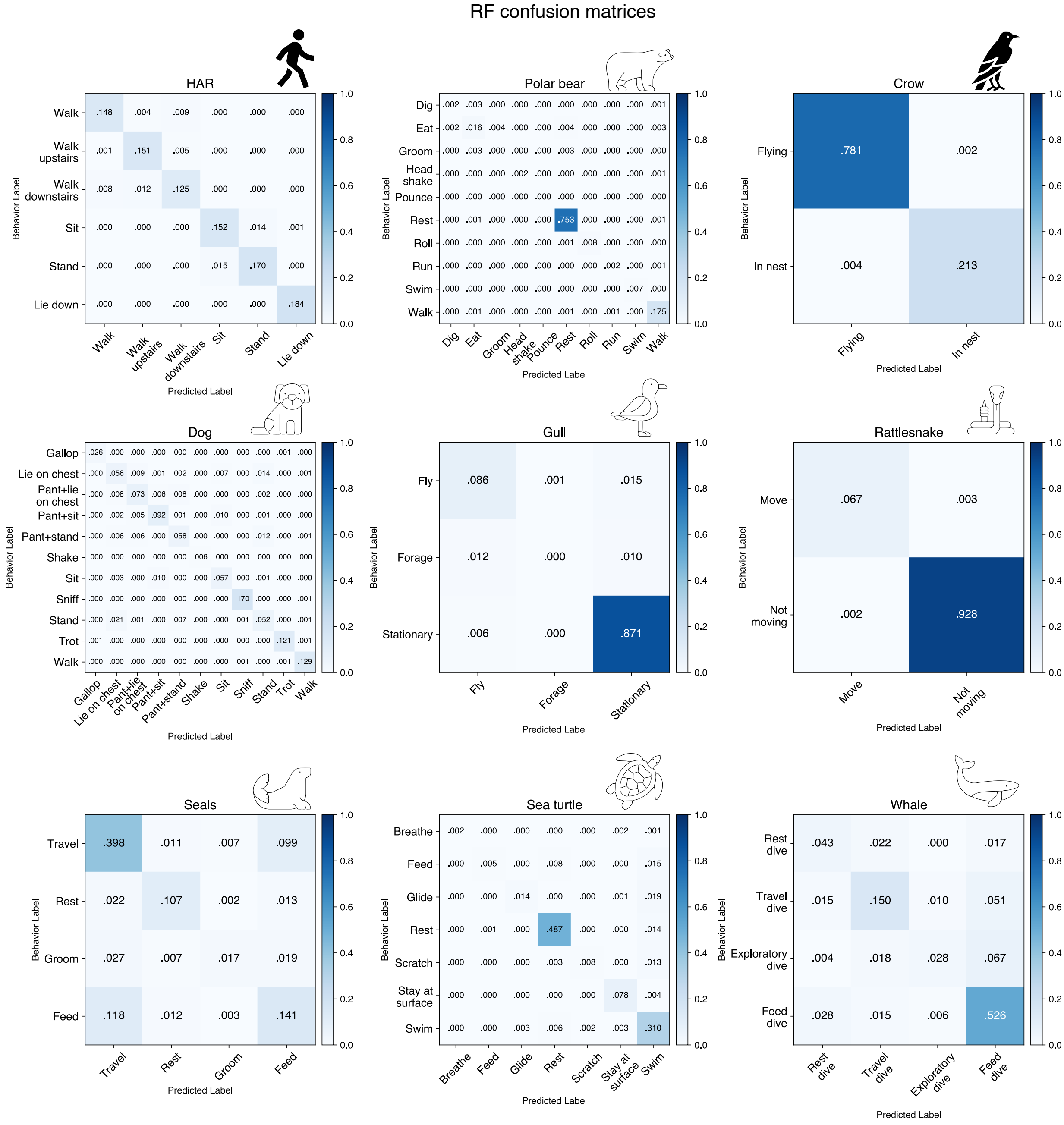

Figure S27: Confusion matrices for RF predictions versus behavioral labels, for all nine datasets in BEBE. Numbers represent the fraction of total labeled data. Computed for data taken from the test sets of the four cross validation steps that were not used for hyperparameter selection. Confusion matrices for the other experiments can be found on the Zenodo data repository.

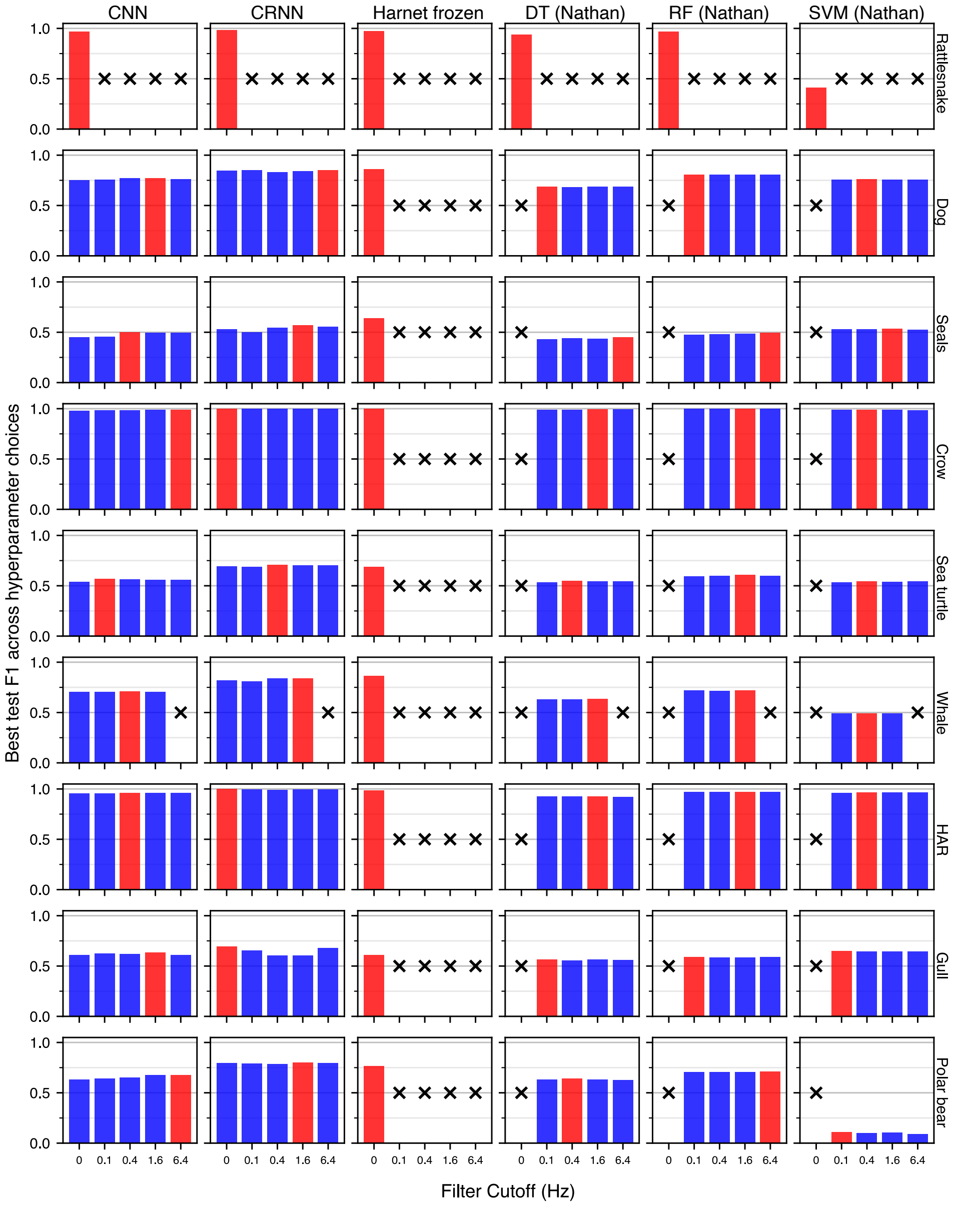

Figure S28: Hyperparameter optimization of low frequency acceleration cutoff frequency. The y-axis indicates the best F1 score on the test set of the fold used for hyperparameter optimization, chosen from all hyperparameter with the same cutoff frequency. A cross marker indicates that this dataset/model pair did not test hyperparameters for the cutoff. The hyperparameter chosen was not consistent within a dataset. Most models do not show large performance variation based on this hyperparameter.

Model	Dataset	#classes	Spearman correlation	p-value
Harnet frozen	HAR	6	0.943	0.017
Harnet frozen	Polar bear	10	0.952	<0.001
Harnet frozen	Crow	2	1.000	1.000
Harnet frozen	Dog	11	0.973	<0.001
Harnet frozen	Gull	3	1.000	0.333
Harnet frozen	Rattlesnake	2	1.000	1.000
Harnet frozen	Seals	4	1.000	0.083
Harnet frozen	Sea turtle	7	1.000	<0.001
Harnet frozen	Whale	4	0.800	0.333
CRNN	HAR	6	0.714	0.136
CRNN	Polar bear	10	0.867	0.002
CRNN	Crow	2	1.000	1.000
CRNN	Dog	11	0.982	<0.001
CRNN	Gull	3	1.000	0.333
CRNN	Rattlesnake	2	1.000	1.000
CRNN	Seals	4	1.000	0.083
CRNN	Sea turtle	7	1.000	<0.001
CRNN	Whale	4	1.000	0.083
RF	HAR	6	0.543	0.297
RF	Polar bear	10	0.976	<0.001
RF	Crow	2	1.000	1.000
RF	Dog	11	0.964	<0.001
RF	Gull	3	1.000	0.333
RF	Rattlesnake	2	1.000	1.000
RF	Seals	4	1.000	0.083
RF	Sea turtle	7	0.821	0.034
RF	Whale	4	1.000	0.083

Table S1: Spearman correlation between reduced and full data settings within a dataset, performance for the given model. p-values computed by two-sided permutation test. We note that the small number of classes limits meaningful hypothesis tests, e.g., Rattlesnake and Crow datasets have $p = 1$ because there are only two classes in these datasets.

Disclaimer: Any use of trade, firm, or product names is for descriptive purposes only and does not imply endorsement by the United States Government.

References

[1] Adam T, Griffiths CA, Leos-Barajas V, Meese EN, Lowe CG, Blackwell PG, et al. Joint Modelling of Multi-Scale Animal Movement Data Using Hierarchical Hidden Markov Models. Methods in Ecology and Evolution. 2019;10(9):1536-50.

[2] Berman GJ, Bialek W, Shaevitz JW. Predictability and Hierarchy in Drosophila Behavior. Proceedings of the National Academy of Sciences of the United States of America. 2016 Oct;113(42):11943-8.

[3] Hunter JD. Matplotlib: A 2D graphics environment. Computing in Science & Engineering. 2007;9(3):90-5.
